# Editorial: Antibiotic Alternatives and Combinational Therapies for Bacterial Infections

**DOI:** 10.3389/fmicb.2018.03359

**Published:** 2019-01-18

**Authors:** Sanna Sillankorva, Maria Olívia Pereira, Mariana Henriques

**Affiliations:** Laboratório de Investigação em Biofilmes Rosário Oliveira, Centre of Biological Engineering, University of Minho, Braga, Portugal

**Keywords:** antibiotic alternatives, bacterial infection, probiotic, bacteriophage, anti-persister molecule, biofilm

“The thoughtless person playing with penicillin treatment is morally responsible for the death of the man who succumbs to infection with the penicillin-resistant organism.” As Alexander Fleming predicted in 1945, bacteria have become increasingly resistant to antibiotics. Penicillin resistance was presumably first reported already in 1940 when Abraham and Chain reported that an enzyme from bacteria was able to destroy penicillin (Abraham and Chain, 1940). Every now and then mankind is shelled with news of infections and deaths caused by antibiotic and multiple drug resistant superbugs. This increase of resistance toward commonly in-use antibiotics, due to decades of their use, misuse and abuse, is today a global health concern. Research investments on development of new agents that can fight antimicrobial resistant microorganisms and the advent of antibiotic failure due to bacterial resistance has raised interest in other non-conventional alternative therapies.

This Research Topic gathers some of the latest science around antibiotic alternatives and the effect of combined therapies. The call was launched in July 2017, and open-call papers were submitted until May 2018. This is the editorial article introducing the 20 accepted publications addressing the antimicrobial action of varied agents representing the breadth and scope of research in this topic.

A high number of publications address the antibacterial use of bacteriophages. A mini review by Morozova et al. describes the main outcomes of English and Russian case reports regarding bacteriophage use in infected wounds, burns and trophic ulcers. The antimicrobial assessment of bacteriophage therapy include *in vitro* testing toward biofilms of *Klebsiella pneumoniae* isolated from diabetic foot patients (Taha et al.), *Staphylococcus aureus* biofilms (Kumaran et al.), and their combined use with honey to control dual species biofilms of *Pseudomonas aeruginosa* and *Escherichia coli* in an *ex vivo* wound model (Oliveira et al.). Overall, bacteriophages were able to decrease bacterial loads and destroy biofilm structures. Bacteriophage-antibiotic treatment order was investigated by Kumaran et al. and this greatly influenced the treatment outcome, and bacteriophages always augmented the activity of antibiotics. Ujmajuridze et al. screened cultures of patients planned for transurethral resection of prostate, adapted the commercial Pyo bacteriophage preparation to target the main species identified (*S. aureus, E. coli, Streptococcus* spp., *P. aeruginosa*, and *Proteus mirabilis*), administered the preparation via intravesical delivery in nine patients, and observed bacterial decrease in six of the nine patients treated. *In vivo* use of a purified bacteriophage capsule depolymerase to treat *E. coli* infections in a mouse thigh model was also studied (Lin et al.). In this work, the authors show that *E. coli* infections, usually lethal to mice, were effectively treated with an enzyme dose of 20 μg per mouse; however this effect was enzyme and capsule type dependent.

Three original research articles assessed the use of probiotics such as *Lactobacillus plantarum* or *L. rhamnosus*. Wang et al. describe the diverse roles of *L. plantarum* from enhancing the intestinal barrier function, inducing the secretion of antimicrobial peptides that protect against pathogens, improving the gut bacterial ecology and barrier function in weaned piglets. The efficacy of *L. plantarum* in preventing enterotoxigenic *E. coli* growth and inhibiting its adhesion to a porcine intestinal epithelial cell line was also assessed (Wang et al.). *L. rhamnosus* was reported to reduce the adhesion of *E. coli* to bovine mammary epithelial cells devoid of the caspase recruitment domain by supressing the NLRP3 and NLRP4 inflammasomes and inhibiting *E. coli*-induced cell pyroptosis (Wu et al.).

Defraine and colleagues reported extensive *P. aeruginosa* membrane damage caused by a novel anti-persister molecule (Defraine et al.) and its antibacterial effect together with different classes of antibiotics toward clinically relevant ESKAPE pathogens (Defraine et al.). The molecule used (SPI009) has great potential to inhibit biofilm growth and eradicate both *P. aeruginosa* and *S. aureus* biofilms, and improved nematode survival when tested in *Caenorhabditis elegans* infected with *P. aeruginosa*. Vitamin C was shown to have antibiofilm activity against *Bacillus subtilis* by reducing the extracellular polymeric substance biosynthesis, with cells becoming more susceptible for killing (Pandit et al.).

An original article by Klitgaard et al. identified potential genes that could be suitable as targets for ciprofloxacin potentiating compounds, and found that in targeting the AcrAB-TolC efflux pump and the SOS response proteins RecA and RecC, *E. coli* resistance to ciprofloxacin was reverted in intermediate susceptible strains.

Antibiotic derivatives were reported by Ramchuran et al., who used three teixobactin derivatives to inhibit methicillin-resistant *S. aureus* (MRSA) growth, giving evidence of its dominant binding mode to lipid II. Antibiotic combinations against established *S. aureus* biofilms were also studied in a hollow fiber infection model. However, no beneficial effect of combination therapy compared to the most effective antibiotic was observed, though the addition of the second antibiotic reduced the rise of bacterial resistant to the first drug (Broussou et al.). Anti-MRSA activity using cationic nanostructured lipid carriers combined with antibiotic was evaluated in mice models of cutaneous infection resulting in infection reduction and improvement of skin barrier function and architecture (Alalaiwe et al.). The topical efficacy and safety of chitogel assembled together with an iron chelator and with a novel broad spectrum antimicrobial effectively reduced *S. aureus* biofilms in an *in vivo* sheep model without causing any topical or systemic adverse effects (Ooi et al.). The topical treatment of recalcitrant chronic rhinosinusitis using colloidal silver was assessed through a 10-day program where patients performed rinsing twice daily (Ooi et al.). Despite being safe, the group of treated patients had similar improvement in symptoms and endoscopic scores as those in the control groups and were inferior to culture-directed oral antibiotics. Tran et al. used an antineoplastic mitotane, that permeabilize the outer membrane of *P. aeruginosa, Acinetobacter baumannii* and *K. pneumoniae*, to exert greater effect to a novel polymyxin, and reduce the emergence of antibiotic-resistant phenotypes.

Three branched RRWQWR-based cationic peptides were designed, synthesized and evaluated revealing higher antibacterial activity against clinically relevant pathogens than the reference peptide (Vega et al.).

We hope that you enjoy reading this Research Topic and find it a useful reference for the state of the art in the emerging field of antibiotic alternatives.

## Author Contributions

All authors listed have made a substantial, direct and intellectual contribution to the work, and approved it for publication.

### Conflict of Interest Statement

The authors declare that the research was conducted in the absence of any commercial or financial relationships that could be construed as a potential conflict of interest.
